# Application of per-residue energy decomposition to identify the set of amino acids critical for *in silico* prediction of COX-2 inhibitory activity

**DOI:** 10.1016/j.heliyon.2020.e04944

**Published:** 2020-10-07

**Authors:** Neha Chaudhary, Polamarasetty Aparoy

**Affiliations:** a Centre for Computational Biology and Bioinformatics, School of Life Sciences, Central University of Himachal Pradesh, Dharamshala, Himachal Pradesh, 176215, India; b Faculty of Biology, Indian Institute of Petroleum & Energy, Visakhapatnam, Andhra Pradesh, India

**Keywords:** Theoretical chemistry, Cyclooxygenase-2, Diarylheterocyclic compounds, Molecular dynamics simulations, Structure-activity relationship, Per-residue energy decomposition

## Abstract

The enormous magnitude of scientific research carried out in the field of NSAIDs and cyclooxygenases (COXs) is known. They are crucial in pain management. COX-2 inhibitors have evolved over the years; from traditional NSAIDs to isoform-specific. The present study is aimed to identify a cluster of amino acids in the catalytic site whose energy contribution can better explain COX-2 inhibitory activity accurately than the binding energy of the whole protein. Initially, MD simulations (25 ns) and MM-PBSA calculations were performed for 8 diarylheterocyclic inhibitors. Per-residue energy decomposition studies were carried out to elucidate the energy contribution of each amino acid, and their correlation with COX-2 inhibitory activity was enumerated. A cluster of catalytic amino acids whose free energy sum has a high correlation with biological data was identified. The cluster of Gln178, Ser339, Tyr341, Arg499, Phe504, Val509 and Ala513 showed the correlation of -0.60. Further, the study was extended to a total of 26 COX-2 inhibitors belonging to different classes to validate the applicability of the cluster of amino acids identified. Results clearly suggest that the cluster of amino acids identified provide accurate screening method, and can be applied to predict COX-2 inhibitory activity of small molecules.

## 1 Introduction

Non-steroidal anti-inflammatory drugs (NSAIDs) constitute a vital class of drugs that inhibit cyclooxygenases (COXs) [1]. COXs are important enzymes in the arachidonic acid metabolism involved in prostaglandin (PG) biosynthesis [2, 3]. The inducible isoform of COX was reported by Needleman, Simmons and Herschman's group, and described as COX-2 [4, 5, 6]. COX-2 has been recognized as a well-known drug target because of its well-characterized role in inflammatory disorders and various cancers [7, 8, 9].

Traditional NSAIDs suppress the activities of both isoforms; the constitutive cytoprotective COX-1 and the inducible COX-2. This led to adverse GI toxicities [10, 11, 12]. In order to develop better anti-inflammatory inhibitors with minimum adverse effects, efforts were made by various research communities for the development of selective COX-2 inhibitors [13, 14, 15]. The structural differences between COX-1 and COX-2 at the active site were exploited [16] for the same. Mutagenesis experiments illustrated that single amino acid substitution i.e. Ile to Val509 (Val523 in PGHS-1 numbering) in the COX-2 enzyme is crucial for its selectivity [17]. The COX-2 binding site is extensively studied by various research groups and it has been found that His90, Arg120, Tyr355, and Glu524 form a hydrogen bond network at the entrance of the binding site, known as gate residues [18]. Other amino acids such as Arg513, Gln192, Phe518, Trp387, Tyr385, Tyr348, Leu359, Tyr355, Leu531, Ser530 and Leu534 (in PGHS-1 numbering) are present towards the interior of the active site [19].

Numerous COX-2 inhibitors have been developed over the years. Structural details of the COX-2 enzyme and the binding mode of its inhibitors were explained previously employing various *in silico* methods like molecular docking and molecular dynamics simulation studies [14, 20, 21, 22, 23, 24]. Generally, COX-2 inhibitors are classified into two major classes on the basis of number of ring structures: (1) Tricyclic or Diarylheterocyclic compounds which possess two proximal diaryl moieties linked to a central heterocyclic or carbocyclic ring; the compounds in this class mainly differ from one another in the central aromatic ring which can either be 4-membered (cyclobutene), 5-membered (pyrazole, isoxazole, furanone) or 6-membered (pyridine, pyranone) [25]; (2) Non-tricyclic compounds: This class of compounds lacks the central cyclic ring. The acyclic core may contain 2 or 3-membered chain e.g. 1, 2-diarylethenes, acetylenes, and chalcone derivatives [25, 26, 27]. The diarylheterocyclic compounds can be further divided into (a) sulfonamide and (b) non-sulfonamides. It has been reported that –SO_2_NH_2_/SO_2_Me moiety at the *para* position of one of the aryl rings is crucial for selective and potent inhibition of COX-2 [19,25,28]. Celecoxib, polmacoxib, and valdecoxib are sulfonamide containing COX-2 inhibitors, whereas rofecoxib, etoricoxib, and SC-58125 have sulfomethyl group.

Apart from the structural classification of COX-2 inhibitors, they may be segregated into four groups on the basis of their selectivity index (SI) (ratio of COX-1 IC_50_/ratio of COX-2 IC_50_). Group 1 NSAIDs suppress COX activity with little selectivity e.g., aspirin, ibuprofen, diclofenac, indomethacin, naproxen, and piroxicam. Inhibitors in Group 2 show 5–50 fold COX-2 selectivity such as celecoxib, etodolac, meloxicam, and nimesulide. Group 3 consists of inhibitor with >50 fold selectivity like rofecoxib, NS-398, and valdecoxib, whereas Group 4 includes those NSAIDs which are weak inhibitors of both isoforms (5-aminosalicylic acid, sodium salicylate, nabumetone, and sulfasalazine) [19].

MM-PBSA Molecular Mechanics-Poisson Boltzmann Surface Area/MM-GBSA (Molecular Mechanics-Generalized Born Surface Area) approaches have become an integral part of structure-based drug design strategies and are being widely employed by various researchers [29, 30, 31, 32].

Earlier we have applied molecular dynamics simulations, and various *in silico* methods for COX-2 and other enzymes [14, 33, 34, 35, 36, 37]. MD simulations and per-residue decomposition studies were performed in order to identify crucial amino acid residues for COXIB binding at the COX-2 active site [38]. In continuation of our previous work, in the present study, we performed MD simulations on an extended set of COX-2 inhibitors in order to identify a group of amino acids whose cumulative free energy can be used to predict COX-2 inhibitory activity. Molecular docking, detailed Structure-activity relationship (SAR) studies and molecular dynamics simulations methods were employed. Per-residue free energy decomposition analysis was also included to elucidate the individual contribution of amino acids involved.

## 2 Materials and methods

### 2.1 Inhibitor data set

**Figure 1:**
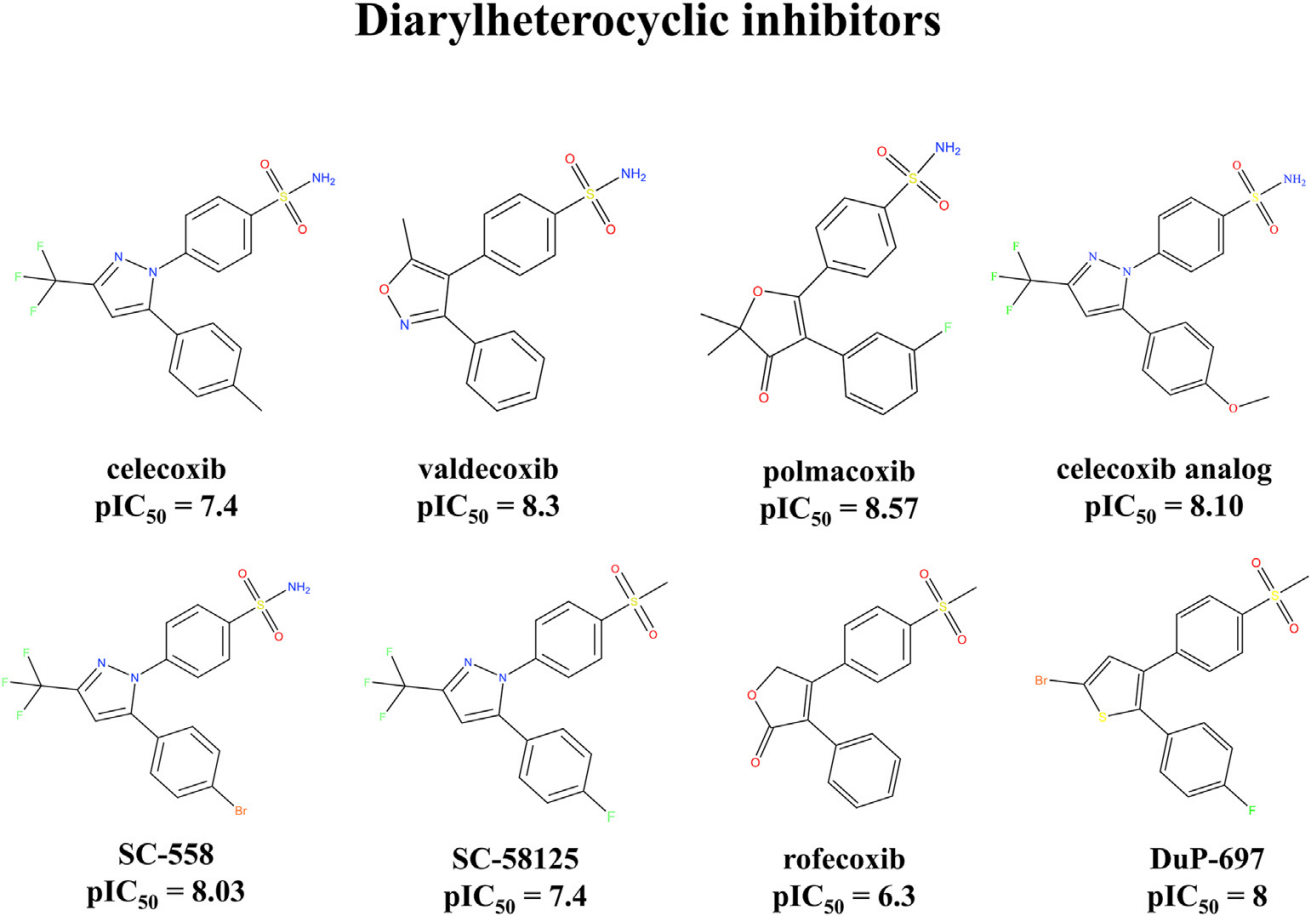
Structures of diarylheterocyclic compounds considered in the study.

**Figure 2:**
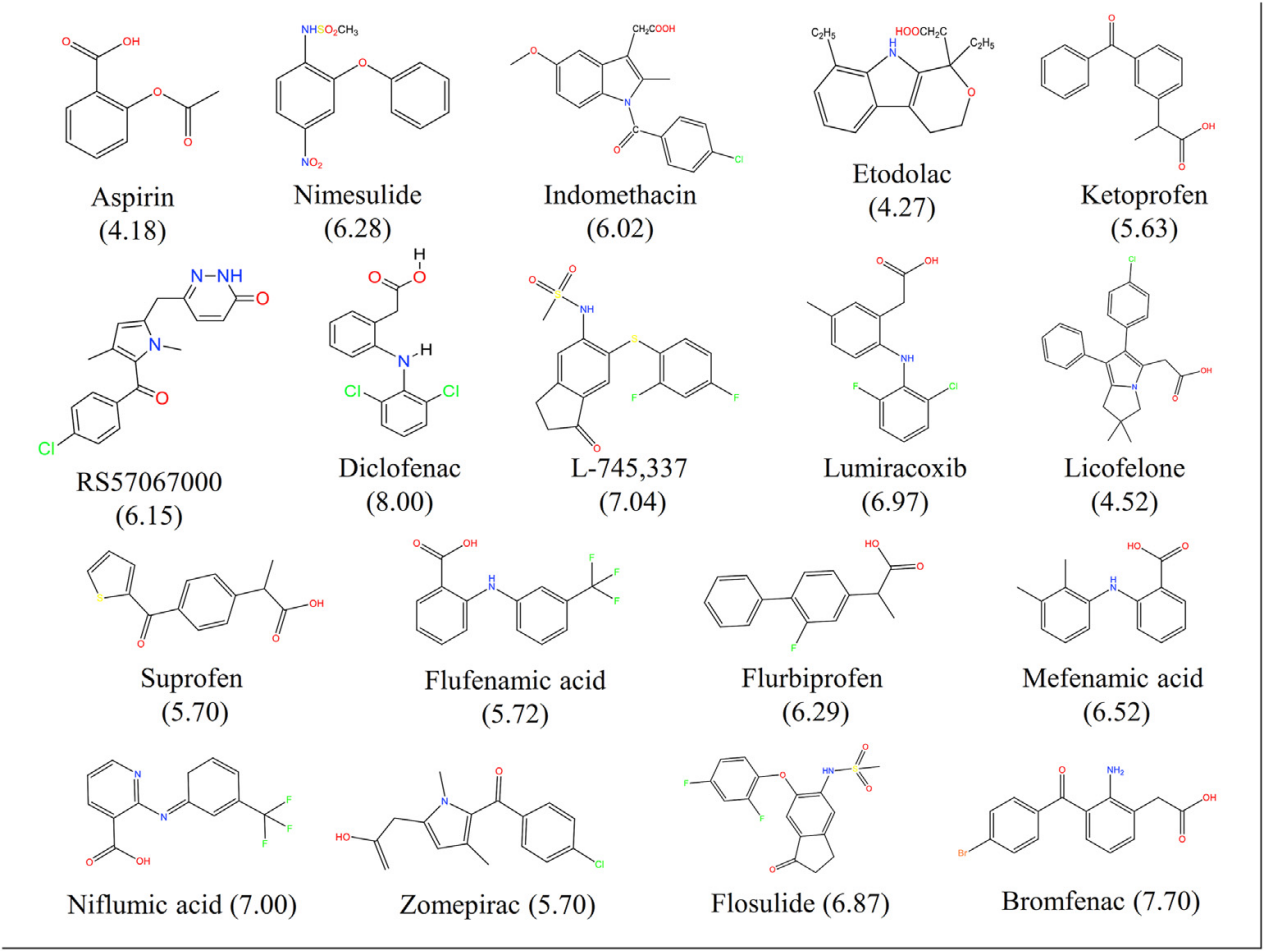
Structures of other inhibitors considered in the study.

A dataset of 26 COX-2 inhibitors belonging to different classes was considered in the study. Among these 26 compounds, 8 belong to the diarylheterocyclic class of compounds, 18 compounds belonged to different classes and were randomly selected from the literature. The diarylheterocyclic inhibitors considered were celecoxib, polmacoxib, valdecoxib, SC-558, celecoxib-analog, rofecoxib, DuP-697, and SC-58125. The chemical structures of the considered inhibitors were given in Figure 1 and Figure 2 with their corresponding pIC_50_ values. The structures of the inhibitors were obtained from the PubChem database [39]. The RED server was used to optimize the structures (RED server uses the Hartree-Fock method for optimization) [40, 41, 42, 43, 44]. Out of the 26 inhibitors discussed in the current manuscript, 3 were studied and reported earlier [38]. They are included and discussed in the current study for better understanding and representation.

### 2.2 Preparation of protein-ligand complexes

**Figure 3:**
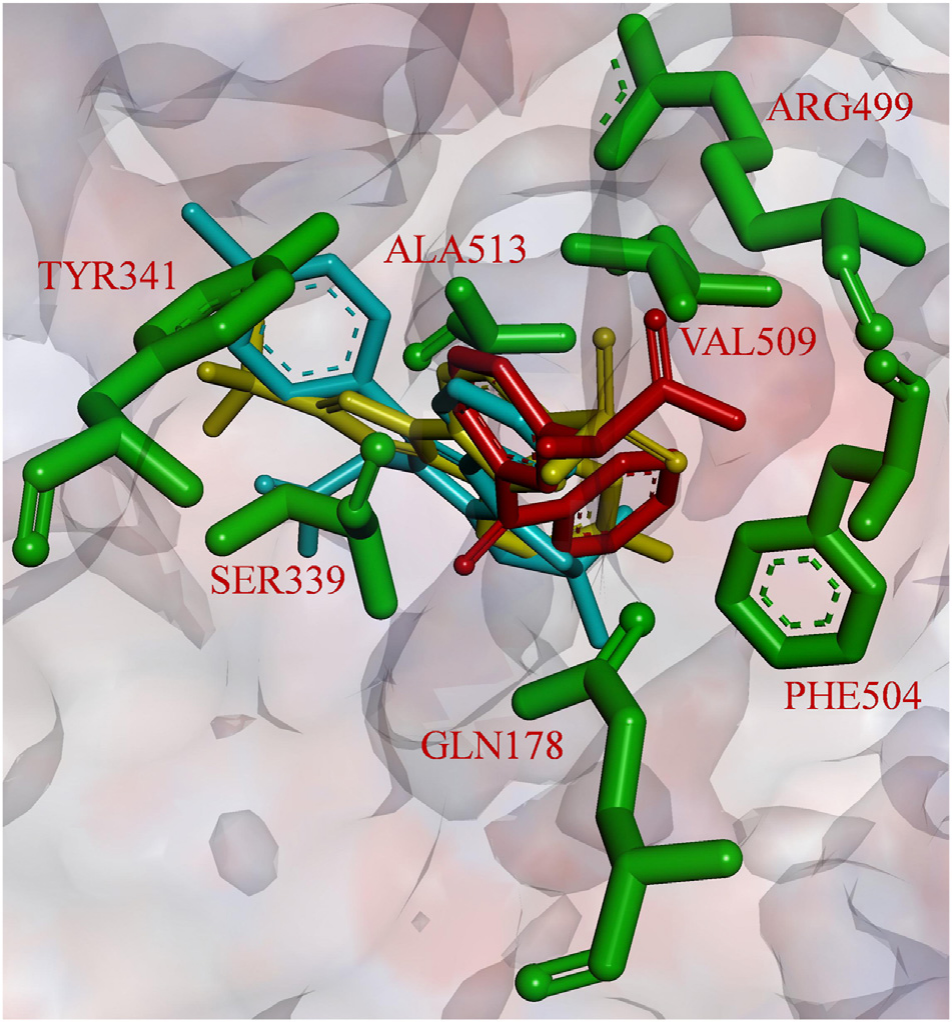
Structure of docked inhibitors at the active site of COX-2; celecoxib (yellow), licofelone (cyan) and ketoprofen (red).

The structure of COX-2 was obtained from the protein data bank (PDB ID: 3LN1). It is a co-crystallized structure of COX-2 and celecoxib. Protein-inhibitor complexes for the other compounds were prepared by implementing molecular docking. The structure of docked celecoxib with two other inhibitors belonging to different scaffolds at the active site of COX-2 is given in Figure 3. The Molecular docking and simulation procedures followed are the same as discussed in our previous work [38], but for a better understanding of the readers, the same is presented in detail here.

### 2.3 Molecular docking

The protein-ligand complexes were prepared using AutoDock [45]. Active site residues were obtained from the bound celecoxib. The accuracy of AutoDock in the prediction of ligand conformation was assured using the re-docking procedure (explained in [38]).

100 docking poses for each inhibitor (with a population size 1000) were calculated. Kollman united atom partial charges, AutoDock atom types and polar only hydrogen atoms were taken into account while preparing protein. Empirical scoring function and Lamarckian genetic algorithm were used for ligand conformational search. All other parameters for docking were set to their default values. The interactions of the best energy ranked conformations were analyzed using Accelrys DS visualizer [46]. The prepared complexes were used as starting structures for further energy minimization and MD calculations.

### 2.4 Energy refinement and molecular dynamics (MD) simulations of the complexes

GROMACS [47, 48] was used in the study. GROMOS96 53a6 [49] force field was employed. Ligand parameters and topology were generated using SwissParam [50]. spc216 [51] water model was used to solvate each system using a cubic box having an edge length of 10 Ǻ. Each system was then neutralized by adding counter-ions. Long-range interactions were treated using Particle mesh Ewald (PME) [52]. Leap-frog integrator [53] was used for MD simulations.

Periodic boundary conditions (PBC) were considered while minimization. Two successive steps of energy minimization were performed using GROMACS. In the first step, 3000 minimization steps were carried out using a steepest descent method. In the second step, 5000 steps of minimization were performed using a conjugate gradient method. The energy step size was set to 0.001 nm in both cases. Each system was then heated from 0 to 300 K for 100 ps in NVT (constant Number of atoms, Volume, and Temperature) ensemble. In NPT (constant Number of atoms, Volume, and Pressure) ensemble, each system was equilibrated using a constant pressure of 1 bar for 100 ps with a time step of 2 fs per step. The coordinates obtained after equilibrating the system were used for MD simulations (25 ns) for each system.

### 2.5 Binding free energy and per-residue decomposition studies

The binding energies for all the systems were calculated using the MM-PBSA method (developed by Srinivasan *et al.* [54]). MM-PBSA method combines the molecular mechanics and continuum solvent models and is explained in our previous report [38]. A total of 250 snapshots from each simulated trajectory of 25 ns were extracted evenly after every 100 ps. The electrostatic energy, van der Waals energy, and polar solvation energy contributions were calculated using Adaptive Poisson-Boltzmann Solver (APBS) [55, 56]. Solvent-accessible surface area (SASA) was used to approximate the non-polar energy contributions. A value of 0.5 Ǻ and 1.4 Ǻ was set for grid spacing and probe radius (for SASA estimation). The solvent dielectric constant was set to 80, whereas the solute dielectric constant was set to 2.

Further, per-residue decomposition analysis was performed to obtain the energetic contribution of the amino acids involved in inhibitor binding. Binding free energy decomposition was performed using the g_mmpbsa tool. This tool decomposes the overall binding energy of the protein-ligand complex [57]. Python scripts “MmPbSaStat.py” and “MmPbSaDecomp.py” were employed for MM-PBSA calculations and individual contribution of different amino acids. In our previous study, all the amino acids i.e., His75, Arg106, Gln178, Leu335, Leu338, Ser339, Tyr341, Leu345, Leu370, Tyr371, Trp373, Arg499, Ala502, Ile503, Phe504, Met508, Val509, Glu510, Gly512, Ala513, Ser516 and Leu517 (present within 8 Ǻ of the active site) were considered. In the present study, Gln178, Val335, Leu338, Ser339, Tyr341, Tyr371, Arg499, Ile503, Phe504, Val509, Ala513 and Ser516 were considered. These amino acids formed interactions consistently with most of the inhibitors. Correlation studies between pIC_50_ values of these inhibitors and per-residue decomposition energies were also carried out. Amino acids showing negative correlation were selected for further study.

### 2.6 Identification of a cluster of amino acids to estimate the ligand-binding affinity

**Figure 4:**
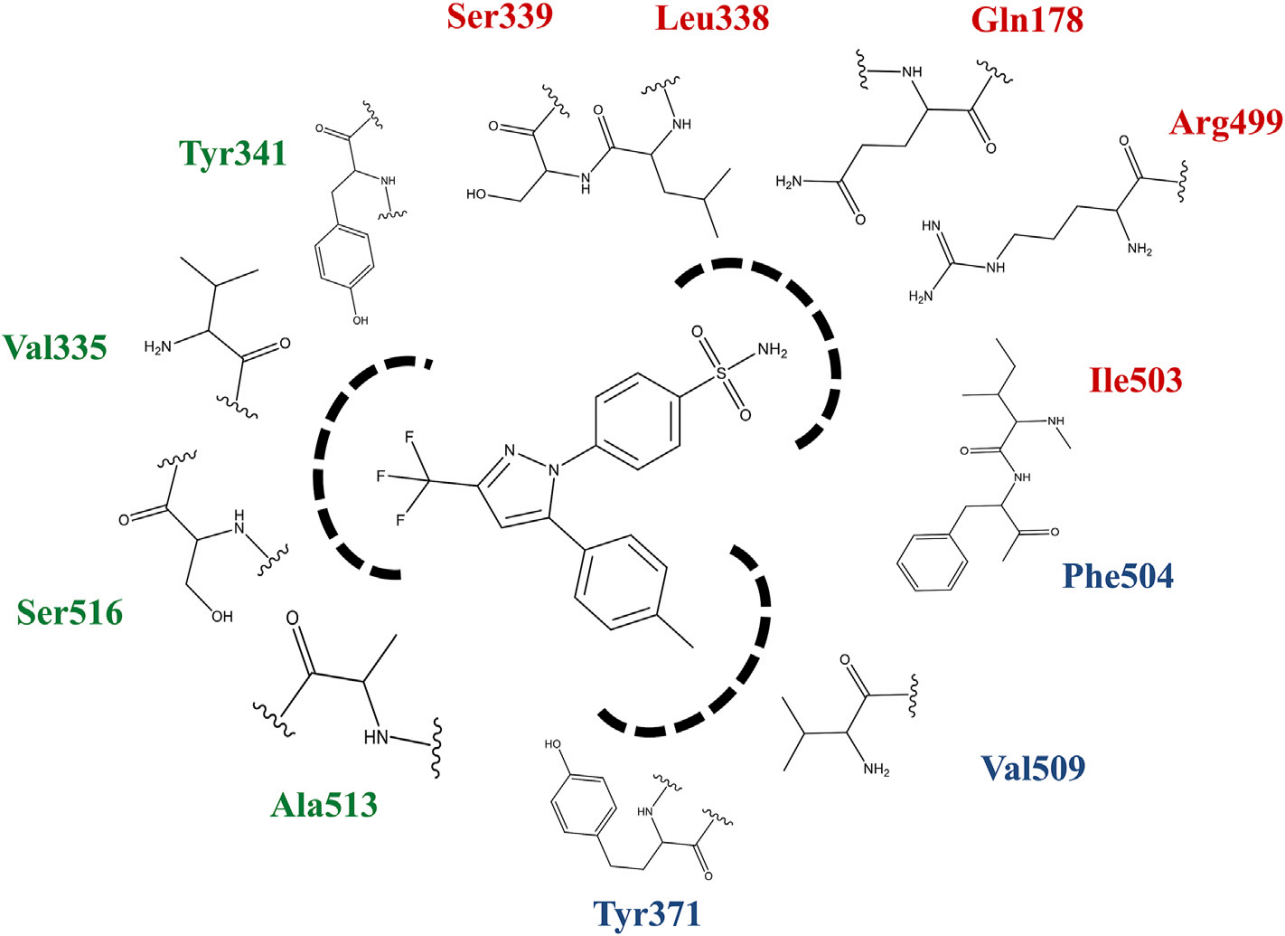
Amino acid sites identified on the basis of interacting atom(s) of the inhibitor, the blue color represents Site-1, red color represents Site-2 and green color represents Site-3.

Site points are points in the active site of a drug target (adjacent to various interacting groups of amino acids) which can be occupied by an inhibitor for favorable binding [58, 59]. The potential of diarylheterocyclic compounds against COX-2 is well known. There are many inhibitors of COX-2 derived from this scaffold. The substituents on these aryl ring hugely contribute to their varied activity. Thorough SAR analysis was performed and three sites (Site-1, Site-2, and Site-3) were identified as shown in Figure 4. Amino acids contributing to the site points corresponding to the substituents at the para position of the phenyl ring (at 3-position) constituted Site-1. For example, hydrophobic –CH_3_ group present in celecoxib and –OCH_3_ in celecoxib analog. Site-2 included the amino acids interacting with sulfonamide/sulfomethyl moiety at the para position of another phenyl ring. Amino acids forming interactions with the central 5/6 membered ring and their functional groups were taken as Site-3. Among all the amino acids present at Site-1, 2 and 3 the ones which have a negative correlation with pIC_50_ were taken. The cumulative binding energy of these amino acids was computed and correlated with biological activity. All the calculations were performed initially for 8 diarylheterocyclic compounds and further extended in 18 other inhibitors. The correlation obtained was compared with that of MM-PBSA energies.

## 3 Results and discussion

In the present work, a variety of COX-2 inhibitors belonging to different structural classes were considered. Molecular docking, molecular dynamics simulations, and MM-PBSA based binding energy calculations were performed.

### 3.1 Docking studies and interaction analysis

After molecular docking, detailed interaction analysis was performed to get insights into the amino acids involved in various interactions.

### 3.2 Molecular dynamics simulations and binding free energy calculations

MD simulations allow a system to interact flexibly, so the dynamics of protein-ligand complexes can be monitored throughout the simulation in order to check protein flexibility and any other conformational changes. MD also facilitates to develop atomistic insights while explaining binding mechanisms. In order to observe the behavior of various inhibitors at the active site of COX-2 during simulation, different properties from simulated trajectories like energy profiles, stability, residue fluctuations, and energy contributions were analyzed.

### 3.3 Interaction analysis after MD simulations

**Figure 5:**
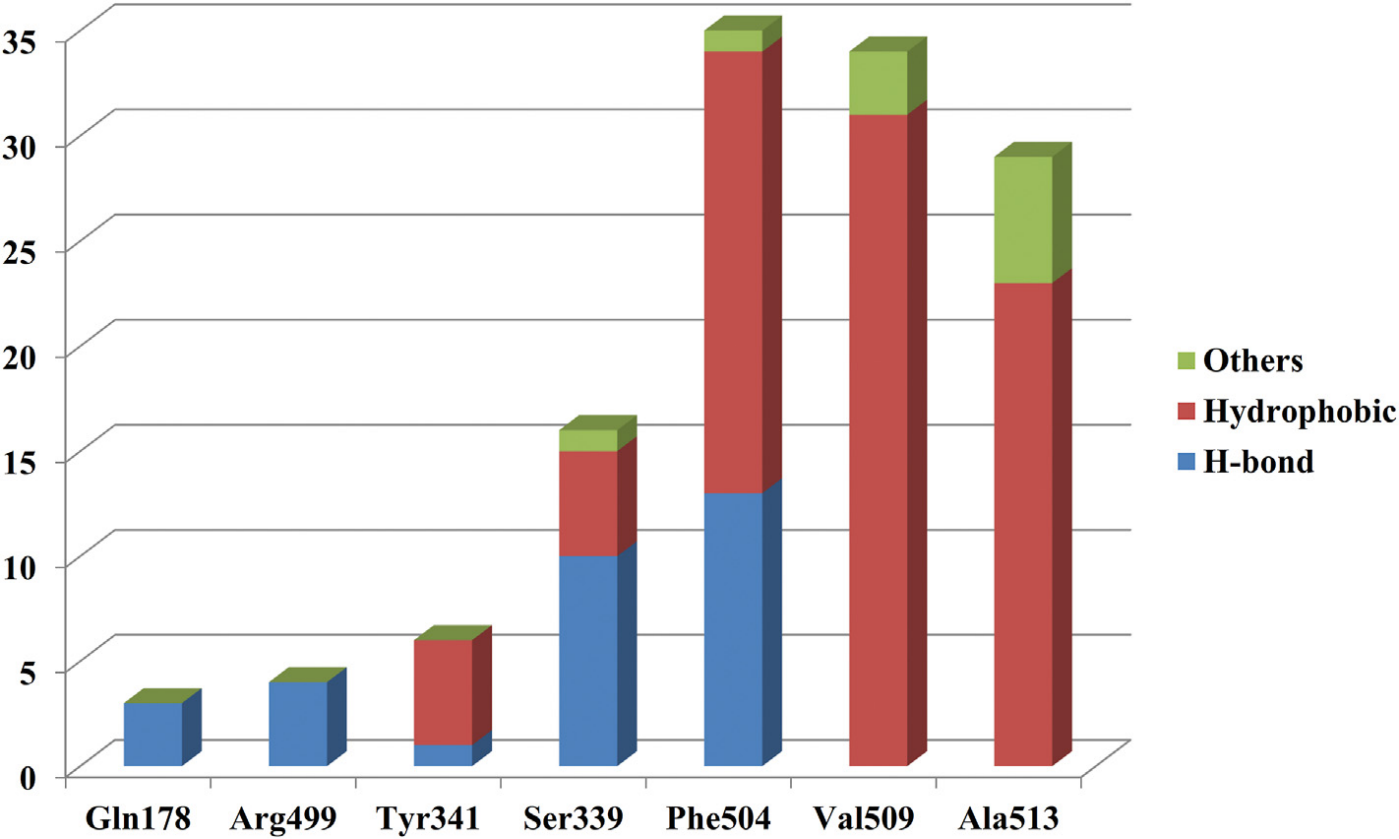
Contribution of selected amino acids in establishing different interactions with various inhibitors.

In order to understand the interactions at the active site of COX-2 all the amino acids present in the 8 Ǻ radius (discussed in the material and method section) were considered in the present study. Detailed interactions were studied and analyzed (Table 1). The interaction analysis provided in-depth understanding regarding the binding mechanisms of diverse inhibitors at the COX-2 active site along with further highlighting the importance of amino acids which are consistently involved in the formation of almost every class of COX-2 inhibitor considered. For the present study, amino acids making significant hydrogen bonds and a large number of hydrophobic interactions (pi-alkyl or pi-pi) were taken. We included a total of 12 amino acids as mentioned earlier. To further quantify the contribution of these amino acids towards COX-2 inhibition, MM-PBSA based binding energy calculations and per-residue decomposition energy analysis were performed. The binding free energy calculation experiments were aimed at elucidating the individual energy contributions of thee amino acids towards the overall binding energy and also to estimate the relationship between these energies and experimental biological activities (pIC_50_) of the investigational compounds. The amino acids included Gln178, Val335, Leu338, Ser339, Tyr341, Tyr371, Arg499, Ile503, Phe504, Val509, Ala513 and Ser516. Among these amino acids Gln178, Ser339, Tyr341, Arg499, Phe504, Val509, and Ala513 were showing negative correlation while Val335, Leu338, Tyr371, Ile503, and Ser516 were exhibiting positive correlation with pIC_50_ (Table 2). We selected these 7 amino acids with a negative correlation for further analysis. To understand the reason for significant correlation detailed interactions of these amino acids across the dataset were thoroughly studied and discussed. Gln178 was forming 3 conventional hydrogen bonds with 3 compounds; Ser339 was forming 10 conventional hydrogen bonds, 5 hydrophobic interactions and 1 other interaction in 12 compounds. Tyr341 formed 1 conventional hydrogen bond and 5 hydrophobic bonds with 5 compounds, Arg499 made 4 hydrogen bonds with 4 inhibitors, and Phe504 formed 13 hydrogen bonds, 21 hydrophobic interactions and 1 other interaction with 25 compounds. Val509 showed 31 hydrophobic interactions and 3 others with 25 inhibitors whereas Ala513 formed 23 hydrophobic and 6 other interactions with 19 compounds (Figure 5).

**Table 1:** Various Interactions formed by inhibitors after MD simulations.

S. No.	Ligand	Hydrogen	Hydrophobic				Others (pi-sulfur, amide-pi stacked/halogen)
			Pi-Sigma	Alkyl	Pi-Alkyl	Pi-Pi	
1	Aspirin	Trp373	-	-	Leu338	Phe504	Met508
		Ser516			Val509		Val509
2	Diclofenac	Arg499	Ser339	Val335	Leu338	-	His75
		Phe504	Val509	Leu345			
		Ser339		Leu517			
3	Etodolac	Tyr371	Leu338	Ala513	Trp373	-	-
		Ser516	Phe504	Val335	Leu338		
				Met508	Val509		
4	Indomethacin	Ser339	Val509	Ala513	Trp373	Tyr371	-
		Ser516		Met508	Phe504	Phe504	
					Ala502		
					Val509		
					Leu338		
5	RS57067000	Ser339	Val509	Val102	Tyr371	His75	-
		Tyr371		Leu517	Trp373	Trp373	
		Ser516			Val335	Phe504	
					Ala513		
					Leu517		
					Val509		
					Ala502		
6	Nimesulide	Phe504	-	-	Val509	Phe504	Phe504
		Tyr341			Ala513	Tyr341	Gly512
					Leu338		Ala513
7	Ketoprofen	Tyr371	-	Val509	Tyr341	-	Met508
		Ser516			Phe504		Val509
					Val335		
					Ala513		
					Leu517		
8	L-745, 337	Ile503	Val509	-	Leu338	Tyr371	-
		Phe504				Phe504	
		Ser339					
9	Lumiracoxib	Ser339	-	Ala513	Val509	Phe504	Gly512
		Gln336		Val335	Ala513		Met508
				Leu338			
				Val509			
				Val102			
10	Licofelone	Val330	Val335	Val335	Tyr334	-	-
				Ala513	Tyr371		
				Leu338	Arg106		
				Met99	Ala513		
				Val102	Ile331		
				Leu103	Val335		
				Leu345	Leu345		
					Leu517		
11	Flufenamic Acid	-	Ser339	Leu338	Trp373	-	Met508
			Val509		Phe504		
					Ala502		
					Val335		
					Leu338		
12	Flurbiprofen	Gly505	-	Ala502	Phe504	-	Leu338
					Val335		Ser339
					Ala513		Arg106
					Val509		
13	Suprofen	His337	Val509	Val509	His75	Phe504	-
		Leu338			Tyr341		
					Phe504		
					Leu338		
					Ala513		
					Met508		
14	Mefenamic Acid	Leu338	-	Leu345	Tyr341	Phe504	Met521
				Val509	Val335		
					Ala513		
					Val509		
15	Niflumic Acid	Ser516	-	Leu370	Phe367	Phe504	Leu370
					Val335	Trp373	Gly512
					Leu338		Met508
					Val509		Val509
							Ala513
16	Zomepirac	Phe504	Ser339	Ala513	Tyr341	-	Met508
		Gly505		Val509	Trp373		
				Met508	Val509		
17	Flosulide	Tyr371	-	-	Val509	Tyr371	Leu370
		Ser516			Ala513	Phe504	Met508
					Leu338		
18	Bromfenac	Ser339	Val509	-	Leu338	Phe504	Gly512
		Ile503			Ala513		Ala513
		Phe504					Met508
		Leu338					
19		Gln178	Ala513		Tyr371		
	celecoxib	Leu338	Ser339	Leu370	Phe504	Tyr371	Gly512
		Ser339	Val509		Leu338		Ala513
		Phe504			Ala513		His75
20	celecoxib-analog	Arg499	Ser339	Val335		-	His75
		Phe504	Val509	Leu345	Leu338		
		Ser339		Leu517			
21		Phe504	-	Met508	Tyr371	Phe504	Arg106
	SC-558	Gln178			Phe504		
		Arg499			Val509		
		Ile503					
22		Arg499	-	Ala513		-	Met508
	polmacoxib	Phe504		Val335	Val509		
		Ser516		Leu517			
		Ser339					
		Ile503					
23		Gln178	Ala513	Ala513	Val335	Phe504	His75
	valdecoxib	Phe504		Val335	Val509	Tyr371	Gly512
		Ser516					Ala513
		Ser339					
24		Ile503	-	-	Leu338	-	-
	rofecoxib	Phe504			Val509		
		Ser516					
25		Ile503	Val509	-		-	Ala513
	SC-58125	Phe504			Leu338		
		Leu517					
26		Ile503	Ala513	Ala513	Leu338		
	DuP-697	Phe504		Val335	Val509	-	-
				Leu517	Val335		
					Ala513

**Table 2:** List of amino acids showing positive and negative correlation with the biological activities of the inhibitors.

Amino Acid	Celecoxib	Polmacoxib	Valdecoxib	Celecoxib-analog	SC-558	SC-58125	Rofecoxib	DUP-697	Correlation
	7.4	8.57	8.3	8.1	8.03	7.4	6.3	8	
GLN178	-2.78	-4.06	-4.34	-1.25	-7.09	0.69	1.15	-0.02	-0.62
SER339	-4.41	-5.66	-8.08	-8.51	-6.03	-4.81	-3.91	-4.29	-0.61
TYR341	-3.47	-4.47	-4.03	-5.72	-4.41	-2.10	-1.93	-4.67	-0.79
PHE504	-10.22	-12.88	-13.90	-15.15	-13.76	-13.31	-13.23	-13.46	-0.28
ARG499	-7.01	-6.97	-2.82	-13.00	-7.58	-1.95	-3.32	-1.21	-0.29
VAL509	-11.56	-10.76	-10.93	-13.66	-12.10	-13.54	-11.63	-10.50	0.20
ALA513	-5.78	-4.29	-4.28	-6.77	-5.41	-6.08	-3.76	-6.35	-0.22
VAL335	-4.70	-5.49	-4.89	-6.07	-6.01	-4.15	-6.18	-5.93	0.03
LEU338	-9.22	-8.10	-9.80	-10.33	-9.11	-10.90	-11.86	-9.46	0.80
TYR371	-2.18	-1.21	-2.62	-4.38	-4.00	-3.10	-5.43	-3.19	0.63
ILE503	-5.38	-5.84	-6.38	-4.39	-6.62	-5.36	-8.13	-7.36	0.44
SER516	1.86	-8.44	-5.76	-0.90	1.22	2.53	-9.18	0.87	0.09

Previous pharmacophore studies have shown the importance of hydrogen bonding and hydrophobic or aromatic interactions in the development of selective COX-2 inhibitors. Pharmacophore model generation for 2-(4-methylsulfonylphenyl)pyrimidine derivatives by Shah *et al.* [60] highlighted the importance of hydrogen bond acceptors and donors in the development of selective COX-2 inhibitors. Michaux *et al.* have generated a structure-based pharmacophore model for 16 COX-2 inhibitors and concluded the importance of a H-bond acceptor, an aromatic ring and two hydrophobic groups for the identification of novel candidates [61]. Palomer *et al.* have investigated pharmacophore features which can account for the activity of selective COX-2 inhibitors for diarylheterocyclic compounds. They have clearly shown the importance of aromatic features for the development of potent and specific COX-2 inhibitors for this class [62]. Another study highlights the significance of a hydrogen bond donor/acceptor, a hydrophobic and one ring aromatic feature in the development of a predictive pharmacophore model for COX-2 inhibitors [63].

### 3.4 Correlation studies

MD simulations and MM-PBSA calculations were performed for the 8 diarylheterocyclic group containing inhibitors. The correlation of ΔU_ele_, ΔU_vdW_, ΔSASA/nm^2^ and ΔG_bind_ with pIC_50_ was deduced. They showed correlation of - -0.04, -0.07, -0.27 and -0.27 respectively (Table 3). The units used to describe binding energy values are kJ/mol.

**Table 3:** Correlation of various energy terms with pIC_50_ values.

	pIC_50_	Van-der Waal energy	Electrostatic energy	Polar solvation energy	SASA energy	Binding energy (kJ/mol)
Celecoxib	7.40	-224.66	-134.98	182.56	-20.95	-198.03
Celecoxib-analog	8.10	-267.01	-165.37	183.58	-20.43	-269.24
Valdecoxib	8.30	-223.09	-141.71	158.65	-18.30	-224.45
Polmacoxib	8.57	-227.50	-135.45	166.50	-20.16	-216.61
SC-558	8.03	-264.45	-150.19	167.53	-20.66	-267.77
SC58125	7.40	-257.63	-97.15	159.46	-20.57	-215.89
Rofecoxib	6.30	-236.89	-136.90	167.51	-18.19	-224.48
DuP-697	8.04	-265.36	-54.28	109.36	-19.36	-229.64
		**-0.07**	**-0.04**	**-0.16**	**-0.27**	**-0.27**

The bold values represent the correlation between the pIC_50_ values and the calculated energy terms.

Per-residue binding energy decomposition analysis revealed the contribution of various amino acids towards total binding energy. From the decomposition analysis, the contributions of the consistently interacting amino acids were extracted, and their correlation with experimental activity was deduced. Gln178, Ser339, Tyr341, Arg499, Phe504, Val509 and Ala513 showed correlation of -0.62, -0.61, -0.79, -0.29, -0.28, 0.20 and -0.22 respectively (Table 2). The energies of Val335, Leu338, Tyr371, Ile503, and Ser516 showed a correlation of almost 0, 0.80, 0.63, 0.44 and 0.09 respectively suggesting an inverse relation with biological activity. Among the amino acids Tyr371, Phe504 and Val509 interacting with the phenyl ring and its substituents (Site-1), Phe504 and Val509 were considered further. Among the amino acids present at Site-2, Gln178, Ser339, and Arg499 were used further. For the amino acids corresponding to the –CF_3_ binding region (Site-3), Tyr341 and Ala513 were used further. The cumulative binding free energy of the group of amino acids was computed. It showed a correlation of -0.60 (Table 4).

**Table 4:** Correlation between pIC_50_ values of the inhibitors and sum of per-residue decomposition energies of the final cluster of amino acids.

Sr. No.	Compounds	Correlation
1.	Diarylheterocyclic Comps	-0.60
2.	Other Compounds	-0.47
3.	All Compounds	-0.70

To check the applicability of the method, 18 inhibitors of other classes were selected. The combined energy contributions of these amino acids showed a significant correlation of -0.47 with the pIC_50_ values of the structurally diverse COX-2 inhibitors (Table 4). The negative contribution of these amino acids towards the overall binding energy and significant correlation (between the combined energy terms and the pIC_50_) suggests their role in the effective binding of structurally diverse COX-2 inhibitors.

In the next phase, the energy contribution of the identified amino acids was summed together for the total dataset of 26 compounds considered in the study and their correlation with the pIC_50_ values was computed and a high correlation of -0.70 was observed (Table 4). These results are in accordance with our previous study wherein we performed MD based studies and reported the importance of Gln178, Val335, Ser339, Arg499 and Phe504 in the effective binding of COXIBs at the active site of COX-2 [64].

These results clearly suggest that the energy contributions of these amino acids can be a clear indication of COX-2 inhibitory activity.

## 4 Conclusion

In the present work, MD simulations, MM-PBSA and per-residue decomposition energy calculations were employed to investigate the binding mechanism of diarylheterocyclic and structurally diverse COX-2 inhibitors. The results obtained in each of the studies were thoroughly analyzed and cross-related. Detailed interaction analysis revealed the importance of Gln178, Val335, Leu338, Ser339, Tyr341, Tyr371, Arg499, Ile503, Phe504, Val509, Ala513 and Ser516 in forming a variety of interactions with the inhibitors considered in the present study. Further, their individual energy contributions were deduced using MM-PBSA and per-residue decomposition energy analysis. The individual energy terms for Gln178, Ser339, Tyr341, Arg499, Phe504, Val509 and Ala513 showed a good correlation with inhibitory activity. The cumulative energy contributions for these amino acids showed a good correlation of -0.60, -0.47 and -0.70 for the diarylheterocyclic, structurally diverse and the total dataset of 26 inhibitors (diarylheterocyclic and structurally diverse COX-2 inhibitors). These amino acids are reported as part of the active site in a number of previous reports but their role in effective inhibitor binding is not discussed. The results of the present study highlight the importance of Gln178, Ser339, Tyr341, Arg499, Phe504, Val509 and Ala513 in inhibitor recognition and binding at the COX-2 active site. These amino acids can be targeted for rational drug design targeting COX-2 and can be of significant importance for lead identification and optimization studies.

## Declarations

### Author contribution statement

Neha Chaudhary: Performed the experiments; Analyzed and interpreted the data.

P Aparoy: Conceived and designed the experiments.

### Funding statement

This research did not receive any specific grant from funding agencies in the public, commercial, or not-for-profit sectors.

### Competing interest statement

The authors declare no conflict of interest.

### Additional information

No additional information is available for this paper.
